# Chronic cough caused by choledochoduodenal fistula: a case report

**DOI:** 10.1186/s12890-021-01658-5

**Published:** 2021-09-10

**Authors:** Jiaofei Cao, Yue Hu, Shaojun Jin, Fei Chen, Li Li, Huaqiong Huang

**Affiliations:** 1 Department of Internal Medicine, The Second Affiliated Hospital of Medical School, Zhejiang University, Hangzhou, Zhejiang China; 2 Key Laboratory of Respiratory Disease of Zhejiang Province, Department of Respiratory and Critical Care Medicine, Second Affiliated Hospital of Zhejiang University School of Medicine, Hangzhou, Zhejiang China; 3 Department of Emergency, Zhuji People’s Hospital of Zhejiang Province, Shaoxing, China; 4 Second Affiliated Hospital, School of Medicine, Zhejiang University, No.599 Yanzhou Avenue, Jiande City, Hangzhou City, 311600 Zhejiang Province China

**Keywords:** Chronic cough, Gastroesophageal reflux disease, Choledochoduodenal fistula, Gastroesophageal reflux-induced cough, Case report

## Abstract

**Background:**

Chronic cough is characterized by cough as the only or main symptom, with a duration of more than 8 weeks and no obvious abnormality in chest X-ray examination. Its etiology is complex, including respiratory disease, digestive system disease, circulation system disease, and psychological disease. Although a set of etiological diagnosis procedures for chronic cough have been established, it is still difficult to diagnose chronic cough and there are still some patients with misdiagnosis.

**Case presentation:**

We present a case of a 54-year-old female patient who had chronic cough for 28 years. Physical examination had no positive signs and she denied any illness causing cough like tuberculosis, rhinitis. Recurrent clinic visits and symptomatic treatment didn’t improve the condition. Finally, gastroscopy identified the possible etiology of choledochoduodenal fistula that was proved by surgery. And after surgery, the patient's cough symptoms were significantly improved.

**Conclusion:**

We report a rare case of chronic cough caused by choledochoduodenal fistula which demonstrates our as yet inadequate recognition of the etiology and pathogenesis. Written informed consent was obtained from the patient.

## Background

With the progress and in-depth understanding of the disease, the original unexplained chronic cough has its corresponding etiological diagnosis, such as cough variant asthma (CVA), eosinophilic bronchitis (EB), gastroesophageal reflux-induced cough (GERC), etc. [1]. In this paper, we share a case of chronic cough caused by choledochoduodenal fistula to enhance our understanding of the complexity of chronic cough.

## Case presentation

**Fig. 1:**
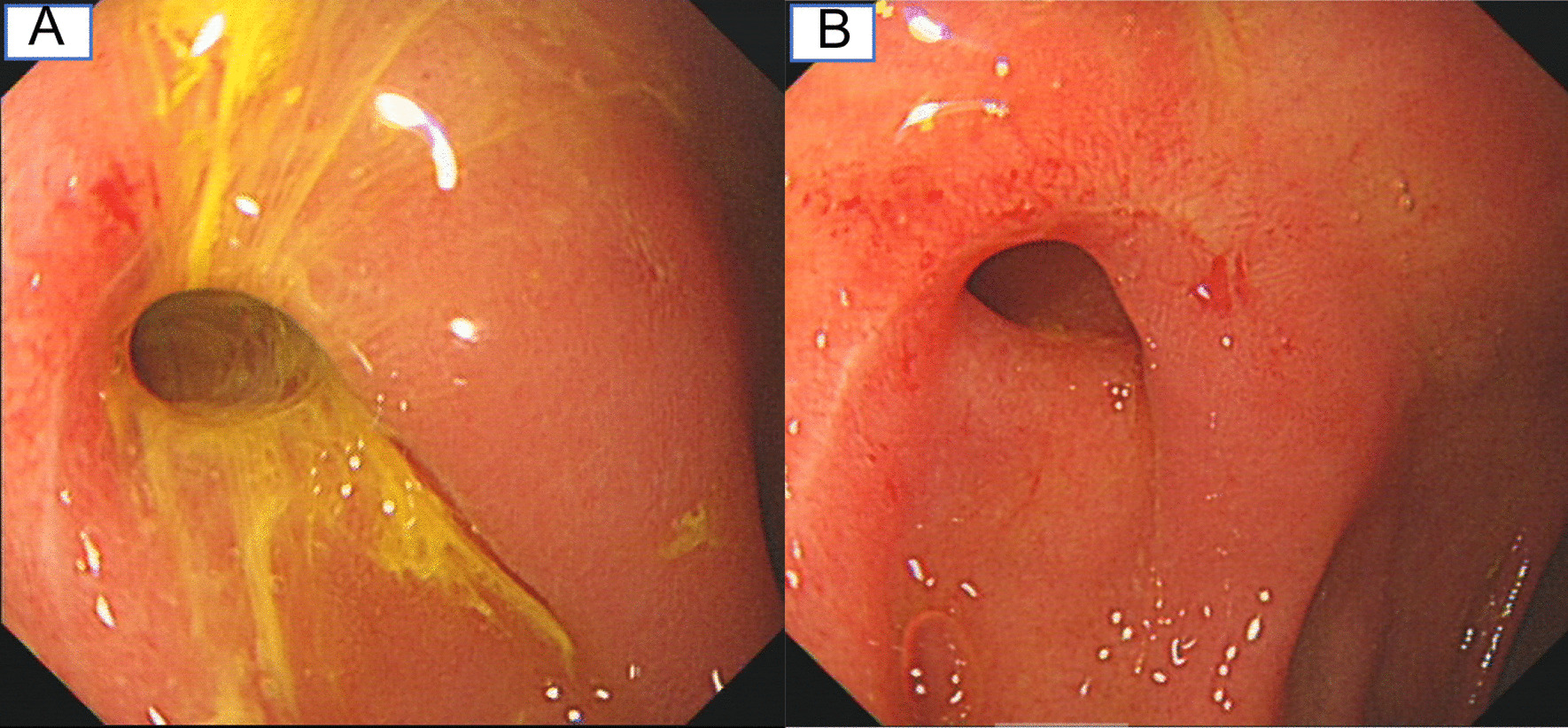
Gastroscope Examination. **A** Gastric pylorus showed massive bile adhesion. **B** Duodenal bulb mucosa is red and white, with patchy erosion, shallow ulcers and bile outflow

A 54-year-old female was admitted to the hospital on December 2020 with the complaint of "recurrent cough for 28 years". This patient developed cough symptoms 28 years ago, without chest distress, shortness of breath, dyspnea, blocked or watery nose, sour regurgitation, belching. She visited the department of respiratory medicine for many years and showed positive bronchial challenge test in 2018 in First Affiliated Hospital of Zhejiang Chinese Medical University, diagnosed as non-severe asthma but the anti-asthmatic treatment had no effect as the patient's symptoms were still repeated. In April 2020 the patient visited the pulmonary specialist clinic in our hospital. She denied tuberculosis, rhinitis, chronic gastritis and gastric ulcer history or hypertension, diabetes but underwent open cholecystectomy 36 years ago and hysteromyomectomy 18 years ago. Physical examination showed no positive signs. Gastroscopy was performed in a local hospital about 1 years ago, and she denied there was some obvious abnormality in the gastroscopy report. In our department, blood tests and allergen were evaluated. The number of Eosinophils and the level of IgE were both normal. Chest CT scan was also ordered in our hospital. The CT scans showed small airway disease in both lungs and fibrous bands in the middle lobe of the right lung. Based on the results of previous and present examinations, the patient was diagnosed with cough variant asthma in April 2020, and treated with montelukast, budesonide formoterol inhaled powder and paracetamol and dihydrocodeine tartrate for about 8 months. The symptoms were relieved but relapsed after drug withdrawal especially of dihydrocodeine tartrate. Because the patient complained of severe coughing and nausea when taking a bus or car and occasional gastrointestinal symptoms such as nausea, vomiting, and acid reflux, the patient was empirically administered with a proton pump inhibitor and symptoms were improved. Therefore, gastroesophageal reflux was considered and the patient was recommended to receive gastroscopy that revealed a fistula in the duodenal bulb with massive bile outflow, and shallow ulcers can be seen around, no abnormality was observed in the descending duodenum (Fig. 1).

**Fig. 2:**
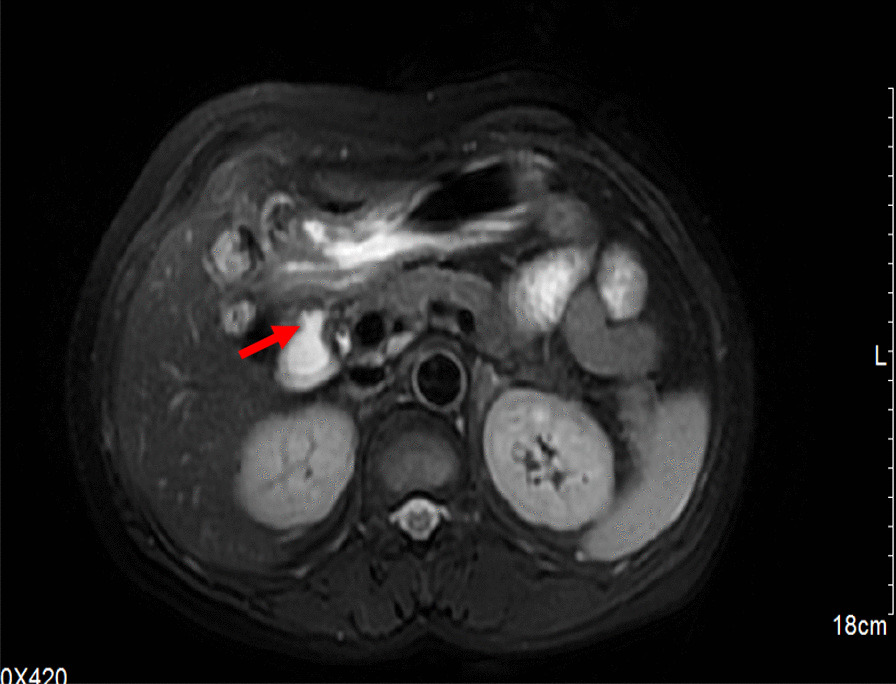
Magnetic resonance cholangiopancreatography presented a choledochoduodenal fistula possibly formed. The lesion is identified with a red arrow

Then, she was admitted to gastrointestinal surgery. Abdominal enhanced CT showed gall bladder absence after cholecystectomy and pneumobilia. Magnetic resonance cholangiopancreatography presented gall bladder absent, intra and extrahepatic bile duct dilatation. The common bile duct is locally adjacent to the descending duodenum, and appears to be connected, considering a choledochoduodenal fistula formed (Fig. 2). The general surgeon considered the patient with operation indications, and the patient was advised to undergo surgery. After the patient completed the cardiopulmonary function tests, excluded surgical contraindications, she received surgical treatment in December 2020. Choledochoduodenal fistula was verified during the operation, and distal gastrectomy + gastrojejunostomy Roux-en-Y reconstruction was performed. After surgery, the patient's cough symptoms were significantly improved.

Now she has been discharged from the hospital with good recovery and without recurring cough.

## Discussion

Chronic cough is a common but intractable disease affecting about 12% general population [2]. Questionnaire survey reports that cough prevalence in many European and American communities is 9–33%. In the United States, patients with chronic cough account for about 10–38% of respiratory outpatients [3]. Not only the number of people suffering from chronic cough but the cost of health resources is enormous. A variety of drugs have been used to treat cough, and inhaled steroids and cough syrups were considered more available. Sales of over counter cough syrup were estimated to be 92.5 million pounds in the UK and 328 million dollars in the US [4]. However, about 60% patients said their symptoms did not improve with any treatment [5].In addition to medications, repeated chest X-rays, chest CT, and bronchoscope have added to the patient's financial burden and greatly affected the patient's quality of life.

According to the etiology of chronic cough in China, cough variant asthma accounts for 32.6%, postnasal drip syndrome is 18.6%, eosinophilic bronchitis is 17.2%, and gastroesophageal reflux-induced chronic cough (GERC) only accounts for 4.6% [6]. However, in western countries, GERC’s proportion is 10–40% [1, 3]. Nowadays, more and more people believe that gastroesophageal reflux disease (GERD), except for postnasal drip syndrome and eosinophilic airway inflammation (asthma, non-asthmatic eosinophilic bronchitis), is one of the most common causes of chronic cough [7, 8].

Gastroesophageal reflux disease (GERD) is a gastrointestinal dysmotility disorder characterized by the reflux of stomach contents into the esophagus and mouth [9], caused corresponding symptoms like intra-esophageal symptoms (acid reflux, heartburn, chest pain) and extra-esophageal symptoms (cough, hoarseness, pharyngitis, etc.). Clinically, the reflux disease questionnaire (RDQ), gastroesophageal reflux disease questionnaire (GERDQ), gastroscope, and 24 h intraoesophageal pH monitoring are used to diagnose GERD [10].However, because the gastroscope and 24 h intraoesophageal pH monitoring are invasive examinations, and the complicated operation of 24 h intraoesophageal pH monitoring, the application of these diagnostics are limited, leading to misdiagnosis of chronic cough etiology to some extent.

Choledochoduodenal fistula (CDF) is a special type of biliary intestinal fistula, exists abnormal channel between the common bile duct and duodenum [11]. It occurs more common in female in China [12], and the risk of this illness is higher when a person has a history of disease of the biliary tract, abdominal pain, fever, jaundice that are prime symptoms of it [13], but some patients have vomiting, diarrhea, and other gastrointestinal manifestations as main symptoms. In this patient, there were no typical symptoms such as abdominal pain, fever, and jaundice, and acid reflux and heartburn were also not obvious. Through gastroscope and magnetic resonance examination, choledochoduodenal fistula was found and finally cured by surgery.

Chronic cough caused by gastroesophageal reflux (GER) can be divided into two subgroups according to the pH value: non-acid GERC and acid GERC respectively. Non-acid GERC is less common than acid GERC, and its diagnosis and treatment have not been standardized yet [14]. Studies have shown that non-acid GERC has similar cough characteristics and cough symptom score to acid GERC, however, has less reflux, heartburn, and lower GERDQ scores compared with acid GERC. Although non-acid GERC responded to standard anti-reflux therapy, cough remission was delayed [15]. This patient presented bile regurgitation under gastroscope, so as to the symptoms of reflux and heartburn were not obvious. It may be one of the reasons why gastroscope has not been performed for decades.

The potential molecular mechanism of non-acid GERC is not fully understood, but it is believed to be related to reflux theory, reflex theory, and airway hyperresponsiveness [14]. The reflux theory states that gastric contents can reflux to the pharynx due to the abnormal structure and function of the lower esophagus, which can be verified by pharyngeal reflux measurement, stimulating cough receptors to cause cough [16]. The reflex theory holds that reflux substances stimulate the esophageal mucosal receptors, activate the cough center to cause the bronchial cough reflex, which is called esophago-tracheo-bronchial reflex theory. In this process of reflection, substance P and other neuropeptides are produced, causing airway inflammation and activating neuropeptide receptors on mast cells, releasing histamine, prostaglandin E2, and other inflammatory mediators, eventually stimulating cough receptors and leading to cough [14]. Besides, in patients with gastroesophageal reflux induced chronic cough, cough threshold is decreased and cough sensitivity is increased [17]. All in all, direct stimulation by reflux or inflammatory mediators by reflex can lead to airway epithelial injury, chronic airway inflammation, and increased airway sensitivity [14]. This may be contributing to positive bronchial challenge in this patient.

In conclusion, the case of choledoduodenal fistula resulting in chronic cough was relatively infrequent. Our article provided a reference for diagnosis among our peers. Generally speaking, the etiology of chronic cough is complex. In addition to paying attention to the medical history and selecting corresponding examinations, the heterogeneity of the disease should be further understood to achieve a better treatment effect.

## Data Availability

All data generated or analysed during this study are included in this published article.

## Abbreviations

CVA: Cough variant asthma
EB: Eosinophilic bronchitis
GERC: Gastroesophageal reflux-induced cough
GERD: Gastroesophageal reflux disease
RDQ: Reflux disease questionnaire
GERDQ: Gastroesophageal reflux disease questionnaire
CDF: Choledochoduodenal fistula
